# The Conditions of Survival of Patients with a SARS-CoV-2 Infection Burdened by Cardiovascular Pathologies in a Temporary Hospital in Gdańsk in 2021 and 2022

**DOI:** 10.3390/jcm14082806

**Published:** 2025-04-18

**Authors:** Dariusz Kostrzewa, Anna Justyna Milewska, Petre Iltchev, Mariusz Kaszubowski, Aleksandra Dorobek, Michał Marczak, Elżbieta Rębas, Aleksandra Sierocka, Remigiusz Kozlowski

**Affiliations:** 1 Department of Management and Logistics in Healthcare, Medical University of Lodz, 90-419 Lodz, Poland; dariusz.kostrzewa@wss.gda.pl (D.K.); petre.iltchev@umed.lodz.pl (P.I.); aleksandra.sierocka@umed.lodz.pl (A.S.); remigiusz.kozlowski@umed.lodz.pl (R.K.); 2 Copernicus Podmiot Leczniczy Sp. z o.o., 80-803 Gdansk, Poland; adorobek@copernicus.gda.pl; 3 Department of Biostatistics and Medical Informatics, Medical University of Bialystok, 15-295 Bialystok, Poland; 4 Professor Tadeusz Bilikiewicz Provincial Psychiatric Hospital, 80-282 Gdansk, Poland; m.kaszubowski@wsp-bilikiewicz.pl; 5 Department of Innovation, Merito University in Poznan, 61-895 Poznan, Poland; michal.marczak@warszawa.merito.pl; 6 Department of Molecular Neurochemistry, Medical University of Lodz, 90-419 Lodz, Poland; elzbieta.rebas@umed.lodz.pl

**Keywords:** COVID-19, SARS-CoV-2, temporary hospital, length of hospitalization, risk of death, effects of age on mortality, cardiovascular diseases in patients with COVID-19

## Abstract

A detailed analysis was carried out on the impact of cardiovascular disease on the risk of death of patients hospitalized at a temporary hospital in Gdańsk during the third and fifth waves of the COVID-19 pandemic (in 2021 and 2022, respectively). **Background/Objectives:** The documentation of 1244 patients was analyzed, of which 701 were hospitalized in 2021 (the Delta variant) and 543 in 2022 (the Omicron variant). The aim of this study was to assess the risk of death of patients with COVID-19 depending on the co-existence of cardiovascular diseases. **Methods:** A model of logistic regression was used to identify the impact of the patients’ age, the coexistence of cardiovascular disease, and the length of hospitalization on the risk of death. **Results:** In 2021, patients were younger (median of 66 years) than in 2022 (median of 74 years), the length of hospitalization was shorter in 2022 (9 days) than in 2021 (11 days), and there was a higher proportion of patients with cardiovascular and respiratory diseases and a medical history of cancer in 2022. The odds of death were also observed to be higher in older patients with cardiovascular disease, particularly those under 73 years of age. In older patients (over 73 years), the odds were paradoxically reduced. **Conclusions:** The age of the patient, cardiovascular disease, and duration of hospitalization affect the risk of death. The Delta variant (2021) was more virulent than Omicron (2022). Cardiovascular disease significantly increases the risk of death in patients with COVID-19. The comprehensive diagnosis and treatment of patients with these conditions may reduce mortality. Further studies are needed on the long-term effects of COVID-19 on the cardiovascular system.

## 1. Introduction

The first case of a coronavirus infection in Poland was confirmed by the Ministry of Health on 4 March 2020. “Patient zero” was an inhabitant of Cybinka (the Lubuskie Province). From then, the first restrictions were introduced in Poland (including the prohibition on the organization of mass events) to prevent the virus from spreading. As a consequence of an increasing number of infections both in Poland and abroad, an epidemiological emergency was declared in the country as of 16 March 2020. International air services were halted, and shops, malls, and entertainment centers were closed. Mass gatherings and events were prohibited. Only groceries, drug stores, and pharmacies could be kept open. In enclosed spaces, an obligation to cover the nose and mouth, maintain social distancing (1.5 m), and disinfect hands was introduced. In the period of 2020–2022, restrictions were loosened or tightened depending on the noted number of infections and fatalities. Currently, restrictions have been removed both in Poland and in many other countries globally. In accordance with the data published by the Ministry of Health as of 25 April 2022 [1], the number of SARS-CoV-2 infections in Poland amounted to a total of 5,991,464 (of which 5,904,430 were new infections, and 5,334,398 patients have recovered). The number of fatalities since 4 March 2020 has reached a value of 115,948. The first reports of COVID-19 came from China, where the mechanisms of disease transmission, incubation periods, and typical clinical symptoms were described much earlier [2,3]. It was established that most of those infected recover on their own within 7 to 10 days. In other cases, the disease process leads to life-threatening complications, including organ failures, septic shock, pulmonary edema, severe pneumonia, and acute respiratory distress syndrome (ARDS) [3]. At the same time, it was indicated that the prevalence and incidence of SARS-CoV-2 increase with age, which is why elderly people and patients with underlying diseases are at a higher risk of a severe course of the disease and even death [4]. It was also noted that the most frequently occurring diseases that increase the risk of a COVID-19 infection included arterial hypertension (21.74%), obesity (20.05%), and diabetes (17.65%) [5,6,7].

A consistent view has emerged in the literature identifying the key risk factors for death due to COVID-19 infection.

1. Age
  - The strongest risk factor is age. The risk of death increases significantly after the age of 65 [8,9].
2. Comorbidities
  - Hypertension (I10–I15) [10];
  - Type 2 diabetes [10];
  - Obesity (BMI ≥ 30) [10];
  - Chronic kidney disease [10];
  - Chronic obstructive pulmonary disease (COPD, J44) [9];
  - Heart failure (I50) [10];
  - Ischemic heart disease (I25) [10].
3. Male Sex
  - Men are statistically more likely to experience severe illness and death [10].
4. Functional and General Condition
  - Tachypnea (rapid breathing) [9];
  - High frailty index (e.g., weakness, dependence on care) [10].
5. Laboratory Markers
  Elevated levels of the following:
  - C-reactive protein (CRP) [9];
  - D-dimer [9];
  - Lactate dehydrogenase (LDH) [9];
  - Interleukin-6 (IL-6) [9].
6. Lack of SARS-CoV-2 Vaccination
  - Unvaccinated individuals had a significantly higher risk of hospitalization and death [8].
7. Socioeconomic Factors
  - Limited access to health care [10];
  - Poor living conditions [10].

The aim of this study is to assess the risk of death of patients treated at a temporary hospital in Gdańsk in the years of 2021 (third wave, dominant Delta variant of the SARS-CoV-2 virus) and 2022 (fifth wave, dominant Omicron variant) due to SARS-CoV-2 infection depending on the coexistence of cardiovascular diseases.

## 2. Materials

The study material was a complete medical dossier of 1244 patients treated at the temporary hospital in Gdańsk during two periods of its operation: the first one lasting from 8 March 2021 to 30 June 2021 (115 days) and the second lasting from 19 January 2022 to 31 March 2022 (72 days). In the first period (in 2021), when the dominant variant of the virus in Poland was the 21A, 21I, 21J mutation (Delta) [11], the hospital treated a total of 701 patients—286 women and 415 men. In the second period (in 2022), when the infection with 21K, 21L, 21M mutation (Omicron) was dominant [12], the temporary hospital treated 543 people, including 279 women and 264 men. Demographic data (gender and age), as well as medical data (the presence of co-existing pathologies with special attention to cardiovascular diseases), were analyzed. The analysis excluded patients who were not eligible for hospital treatment at the temporary hospital for the following reasons:

1. The patient did not consent to hospitalization or withdrew their consent for hospitalization;
2. The patient was transported to another facility directly from the reception point of the temporary hospital due to medical indications, including an acute coronary event, fresh stroke, or required urgent surgical intervention;
3. A lack of complete data in the documentation, enabling the patient to be clearly assigned to a specific study group.

In 2021, there were 3 such situations, and in 2022, there were 13. In every case, the course of SARS-CoV-2 infection in the stage of the hospital admission procedure was defined as mild. Fifteen patients who were disqualified from treatment at the temporary hospital were not included in the analyses. The general condition of each of them was assessed as good and stable. Their contact with the facility was limited solely to their presence at the admission point, followed either by the transfer to another center or return to their place of residence. In the case of one patient, no data regarding the stay or medical services provided could be identified. It was most likely an erroneous entry containing only a name and surname assigned to the temporary hospital.

**Figure 1:**
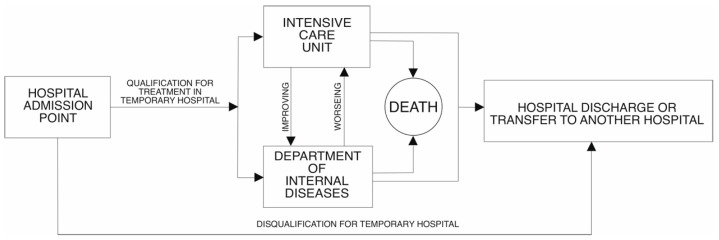
A diagram of the flow of patients to the temporary hospital in 2021.

**Figure 2:**
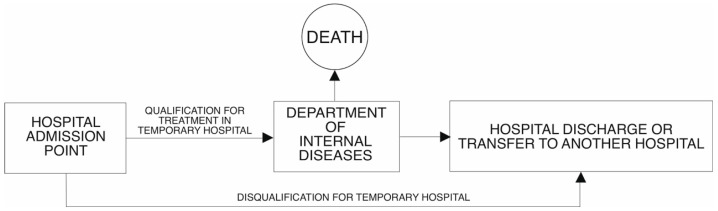
A diagram of the flow of patients to the temporary hospital in 2022.

In the first period of the temporary hospital (in 2021) in its structure, there was an intensive care unit, in which a total of 88 patients were treated (12.5% of all patients admitted). In the second phase (in 2022), the Minister for Health did not require the availability of an intensive care unit in the structure of a temporary hospital. For this reason, patients requiring respirator treatment were transported to another facility. This situation occurred in 10 cases, which represented 1.8% of the total number of patients treated. The flow of patients to the TH in 2021 and 2022 is presented in Figure 1 and Figure 2, respectively.

The analysis of deaths for 2022 also included patients who died in the facility to which they were transported from the TH. In particular, this applies to the intensive care unit for patients with COVID-19 and the Health Care Centre, which continued to care for patients from the TH after they had obtained a negative result of the virus test.

The study was conducted in accordance with the Declaration of Helsinki and approved by the Ethics Committee of the Regional Medical Chamber in Gdańsk (number KB-36/22 on the date of 12 July 2022).

## 3. Methods

According to the general definition adopted by the World Health Organization, death is defined as the permanent, irreversible cessation of functions of the organs essential for life (regardless of the time since live birth), which consequently results in the cessation of vital functions of the entire organism. For clinical purposes, the determination of death is based on the absence of breathing, pulse, pupil response, consciousness, or—in doubtful cases—criteria for brain death (confirmed by specialized procedures). In the analyzed material, no cases of questionable death determination were identified.

A hospital death is defined as the death of a patient occurring during hospitalization, i.e., while the patient is admitted to the hospital, regardless of the cause of death. The key criterion is that the patient was under the care of the hospital at the time of death, meaning they were formally admitted, regardless of their exact location. This study includes all deaths that occurred within the temporary hospital as well as in the intensive care unit for COVID-19 patients located in another facility to which the patient had been transferred. Because this ICU unit is part of the same company managing the temporary hospital, the medical documentation is complete and reflects the continuity of care and treatment.

This study had a basic (fundamental) character with a practical aspect. Its aim was to identify the risk of patient mortality in an early stage of hospital treatment based on data from medical interviews and basic diagnostic tests. The analytical value of the study lay in identifying statistically significant correlations between groups of cardiovascular diseases and the risk of death.

For obvious reasons, this was a retrospective study, based on historical data from completed hospitalizations. The researchers did not interfere with the study conditions in any way but instead conducted statistical analysis of numerical variables (such as patient age, number of diagnoses according to ICD-10, and mortality rate) and qualitative variables (such as gender and diagnoses).

The case–control aspect of the study consisted of comparing the number of deceased patients with a given condition to the number of recovered patients with similar comorbidities.

The collected data are presented in the form of descriptive statistics: for qualitative data, these are the number of observations and the percentage of selected cases; for quantitative variables, these are the number of observations, the median, the lower quartile, and the upper quartile. To investigate the dependence of categorized variables, a Pearson’s chi-square test was used. To compare the independent variables in the absence of a normal distribution, the Mann–Whitney U test was used. Simple models of logistic regression were designated, and a multifactor model was built. The goodness of fit was assessed with the Hosmer–Lemeshow test, and a calibration curve was created. To assess predictive properties, an ROC analysis and a classification table were performed. A statistical significance level of 0.05 was assumed. The analyses were performed using R-Studio (2024.12.0+467), StataSE 18.0 (Stata Corp LLC, Collage Station, TX, USA), STATISTICA 13.3 (TIBCO Software Inc.; Santa Clara, CA, USA) statistical packages, and graphs were created in Microsoft Excel. This study was conducted in accordance with the principles of retrospective analysis on anonymized data.

## 4. Results

For analytical purposes, diagnoses were classified as cardiovascular diseases according to the 10th Revision of the International Statistical Classification of Diseases and Related Health Problems (ICD-10). Cardiovascular diseases were classified in Chapter IX, sections I00 to I99. Diagnoses were based on physicians’ assessments at the end of treatment—either upon discharge or death.

The number of cardiovascular disease diagnoses is summarized in Table 1 below.

**Figure A1:**
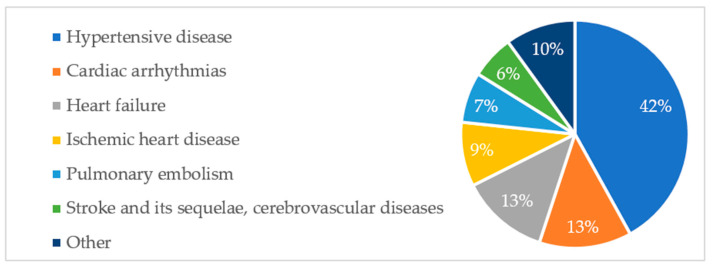
Most frequently diagnosed cardiovascular diseases.

**Figure A2:**
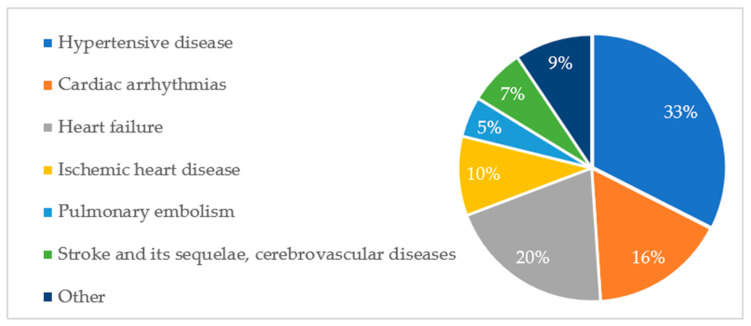
Cardiovascular disease diagnoses among deceased individuals.

Aggregated data show that six diagnosis groups predominated in the study population, accounting for 90% of all cardiovascular pathologies. The percentage share of groups of cardiovascular pathologies is shown in Figure A1. A similar distribution of diagnoses was observed among deceased patients. This is illustrated in Figure A2.

A similar analysis was conducted for respiratory disease diagnoses. Five diagnostic groups predominated, accounting for over 90% of all diagnoses. The data are shown in Table 2.

**Figure A3:**
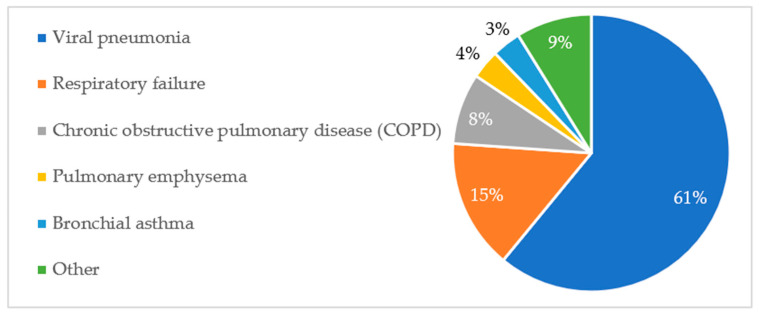
Frequency of specific pulmonological diagnoses.

**Figure A4:**
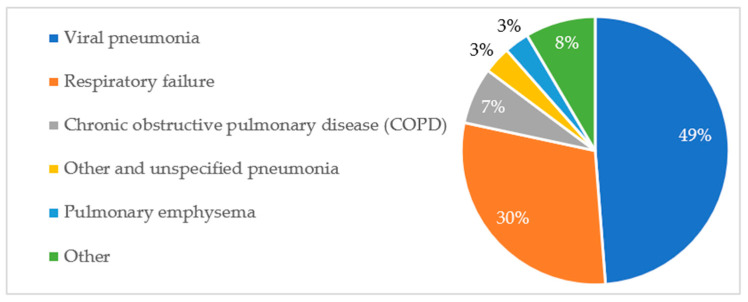
Frequency of respiratory disease diagnoses among deceased patients.

The percentage distribution of respiratory pathologies and their association with patient mortality are presented in Figure A3 and Figure A4, respectively.

It should be noted that the diagnosis of pneumonia was, in the majority of cases, primarily caused by the SARS-CoV-2 virus, while respiratory failure constituted the direct cause of death in all deceased patients. Therefore, it is not possible to consider these diagnoses as factors increasing the risk of death.

Due to the small number of oncological diagnoses and the exclusion of their impact on the mortality risk of patients with SARS-CoV-2, only a numerical summary of the diagnosis groups is presented in Table 3.

**Figure A5:**
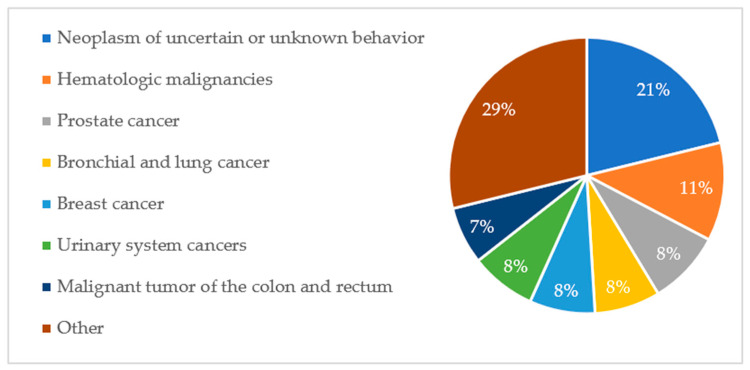
Frequency of specific oncological diagnoses.

**Figure A6:**
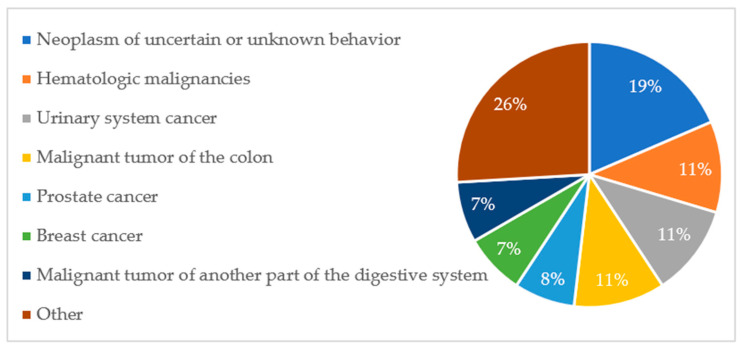
Frequency of oncological disease diagnoses among deceased patients.

The percentage distribution of oncological diagnoses and their contribution among deceased patients are presented in Figure A5 and Figure A6, respectively.

### 4.1. Differences in the Course of Hospitalization Related to the Year

**Figure 3:**
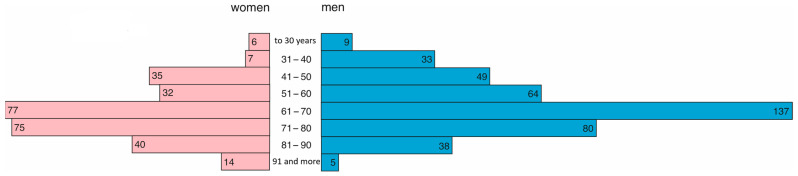
Age and gender distribution in 2021.

In 2021, 286 women and 415 men were treated in the hospital. Women were, on average, 69 years old (Q1 = 56, Q3 = 78), and men were younger and, on average, 65 years old (Q1 = 53, Q3 = 73). This difference was statistically significant (*p* = 0.0001). The age structure is presented in Figure 3.

**Figure 4:**
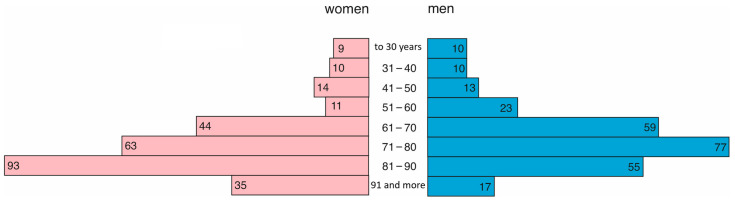
Age and gender distribution in 2022.

In 2022, 279 women and 264 men were treated in the hospital. As in the previous year, women were older (median = 77, Q1 = 67, Q3 = 87) than men (median = 72, Q1 = 63, Q3 = 82). This difference was statistically significant (*p* = 0.0001). The age structure is presented in Figure 4.

The average age of the patient in 2021 was 66 years (Q1 = 54; Q3 = 75), while in 2022, it was statistically higher (*p* < 0.0001) at 74 years (Q1 = 64; Q3 = 85). The length of hospitalization varied between years (*p* < 0.0001). In 2021, the average treatment duration was 11 days (Q1 = 7, Q3 = 15), and in 2022, it was shorter at 9 days (Q1 = 6, Q3 = 13). In 2022, there were statistically significantly more patients with cardiovascular and respiratory diseases and with a medical history of cancer compared to 2021 (Table 4).

### 4.2. Analysis of Hospitalization Ending with the Death of a Patient

The cause of all hospitalizations at the temporary hospital was either an exacerbation or decompensation of a chronic illness, or the onset of subjectively perceived dyspnea in conjunction with confirmed COVID-19 infection. Out of 1244 hospitalizations, 290 ended in death, accounting for 23.3% of admitted patients. Acute cardiopulmonary failure was identified as the direct cause of death in all cases.

Patients who died were, on average, 76 years old (Q1 = 69, Q3 = 86), while those who survived were 67 years old (Q1 = 54; Q3 = 77). This difference was statistically significant (*p* < 0.0001). The average length of hospitalization did not differ between patients who died and survived (median = 10 days, *p* = 0.1468).

Among patients who died, 25.6% had accompanying cardiovascular disease, while among those who survived, the percentage was lower, 18.2% (*p* = 0.0040). A noticeable prevalence of deaths, although not statistically significant, also applied to patients with a respiratory system pathology, an oncological medical history, and those who were treated in 2021 in relation to the year 2022 (Table 5).

### 4.3. Logistic Regression Model

To assess the simultaneous impact of multiple factors on the odds of death of the patient, a logistic regression analysis was used. Simple models demonstrated only an impact of age on the patient’s death (OR = 1.054) and cardiovascular diseases (OR = 1.550). A multifactor model obtained by a backward stepwise regression method included the following variables: age (OR = 1.088), length of hospitalization (OR = 0.979), year (OR = 0.474), cardiovascular disease (OR = 11.557), and interaction of age with cardiovascular disease (OR = 0.967) (Table 6 and Table 7).

**Figure 5:**
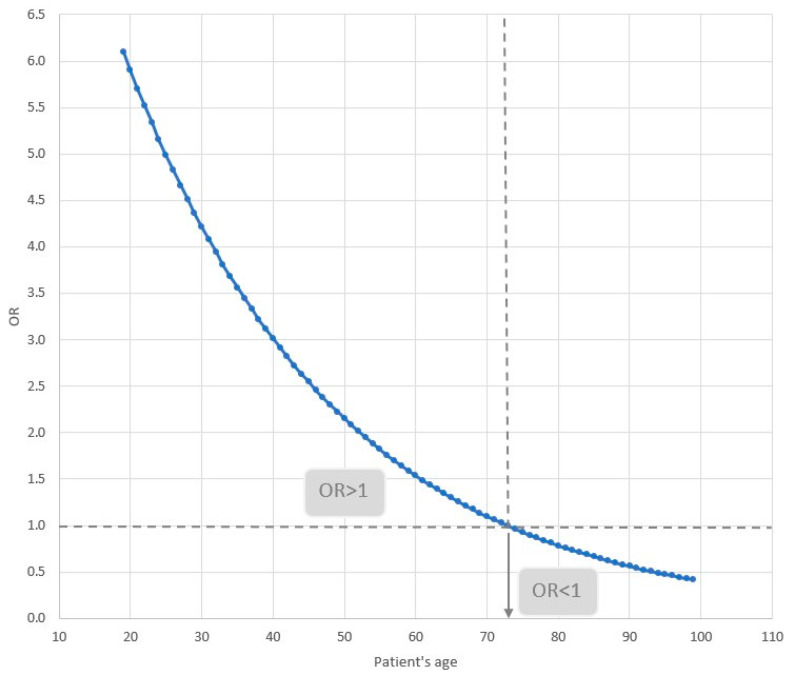
The interaction of odds ratios for the age of the patient. Blue line—relationship between OR and age. Horizontal line—indicates the level of OR = 1, below the line the odds ratio of death decreases, above the line the odds ratio of death increases. Vertical line—indicates the age (73 years), at which the nature of OR changes.

Interpretation of the impact of variables present in the multifactor model on the odds of death of the patient:

- If the patient’s age is increased by 1 year, the odds of death will increase by 8.8%.
- If the hospitalization is extended by 1 day, the patient’s odds of death will decrease by 0.21%.
- The odds of death of a patient treated in 2022 are 2.11 times lower than those of a patient treated in 2021.
- The odds of death of a patient with cardiovascular disease depend on age. This relationship is illustrated in Figure 5. For patients younger than 73 years of age, the odds of death increase, while for those 73 and older, they decrease. For example, for a patient who is 30 years old, the odds of dying with co-existing cardiovascular disease are 4.2; for a patient who is 50, they are 2.2; and for a patient who is 80, they are 0.8.

**Figure 6:**
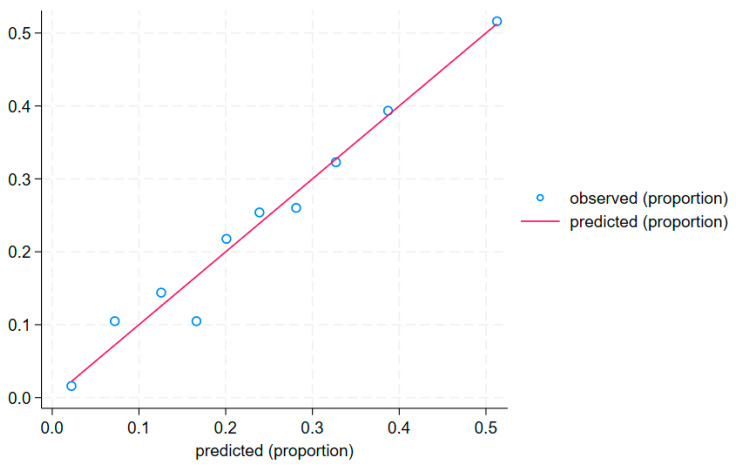
Calibration curve.

The calibration curve (Figure 6) and the Hosmer–Lemeshow test (*p* = 0.759) confirm a good fit of the model to the data.

**Figure 7:**
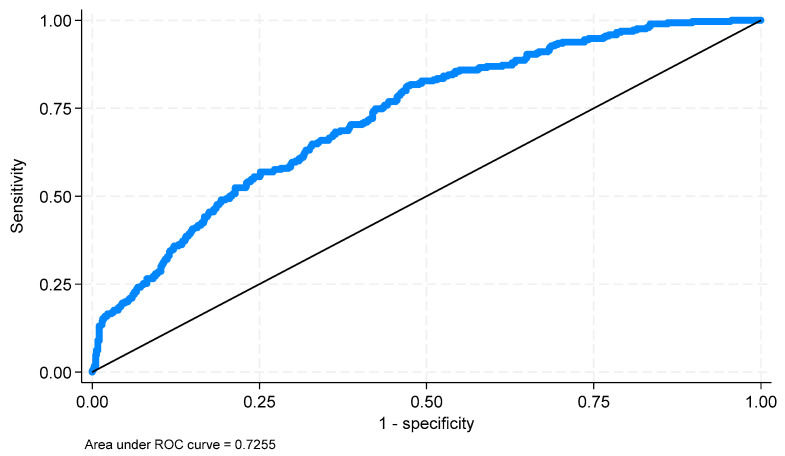
ROC curve for the described survival model.

An ROC analysis was performed to assess the values predicted by the model. The area under the ROC curve (AUC) is 0.7255 (*p* < 0.0001; 95% CI: 0.6935; 0.7574) (Figure 7). For the curve obtained using the Youden index, a cut-off point of −1.343 was determined. The resulting classification (Table 8) was characterized by a sensitivity of 0.817% and a specificity of 0.524%.

## 5. Discussion

The temporary hospital was established due to the rapidly increasing number of patients infected with SARS-CoV-2 who required isolation and inpatient treatment. Because of the rapidly growing number of patients and time constraints, a range of procedural simplifications was implemented. These included, among others, omitting a detailed interview regarding compliance with recommended epidemic-related behaviors. Medical documentation did not include data on mask-wearing or social distancing. As a result, we did not have access to reliable and complete data of this kind. Only in isolated cases was information obtained on issues such as nicotine use. This study focuses on comorbidities accompanying COVID-19 infection, particularly on their significance in increasing the risk of death in infected patients.

The issue of the effectiveness of prophylactic vaccinations has been the subject of many studies and publications worldwide. A clear positive effect of vaccination has been demonstrated in reducing infection rates, ensuring milder disease courses, and decreasing mortality in COVID-19 cases. Multicenter studies from the USA (E. Anderson et al.) indicate a 2.46 times lower risk of death among vaccinated individuals [13]. Similar findings were reported by M. Sayeed and colleagues in Pakistan [14], where the risk of death among vaccinated individuals was 43 times lower compared to unvaccinated patients. Although studies conducted in Russia and Kazakhstan [15] did not confirm the impact of vaccination on mortality, they did highlight a milder course of disease in vaccinated individuals.

It is important to note that our study included only individuals with confirmed COVID-19 infection. Their immunity to the virus had already been compromised, regardless of whether they were vaccinated or not. It should also be noted that the first launch of the temporary hospital in Gdańsk occurred one week before the start of Poland’s general vaccination campaign, which began on 15 March 2021. Prior to that, on 26 January 2021, vaccinations were initiated for people over 60 years of age, and on 12 February 2021, vaccinations became available to patients with chronic diseases. The earliest group to receive vaccinations, on 26 December 2020, was medical personnel, followed by teachers and emergency service personnel on 12 January 2021.

Given the above timeline of the vaccination campaign, at the time the temporary hospital began admitting patients, only a small percentage (approximately 7%) had received at least one vaccine dose. Only about 2% of patients admitted at that time had received both doses. By the end of the first phase of the temporary hospital’s operation in the Pomeranian Voivodeship, only around 50% of the region’s residents had been vaccinated. Precise data are difficult to obtain, due in part to population migration.

The share of older people in the entire studied population is much more pronounced in the year 2022, when the Omicron variant dominated. This observation is consistent with the literature data, in which the dominant view is that this mutation affected more young people, including children [16]. The TH in Gdańsk was designed for the treatment of adults only. The relationship between the patient’s age and incidence of SARS-CoV-2 is confirmed by data from many regions of the world, including China, Italy, Japan, Singapore, Canada, and South Korea [17], where it is estimated that the susceptibility to infection in people under the age of 20 is about half that of adults over the age of 20. A natural consequence of the incidence is mortality, so the risk of death in older people is greater. Extensive meta-analysis suggests that cardiovascular disease (CVD) and its risk factors (hypertension and diabetes) were closely related to fatal outcomes in the course of COVID-19 in patients of all age groups. Although younger patients had a lower rate of co-existing cardiovascular diseases than older patients, the relative mortality risk in young patients with hypertension, diabetes, and cardiovascular disease was higher than in older patients. This does not change the fact that older age is generally a major risk factor for a severe course of COVID-19 [18].

Immunosenescence, or the age-related weakening of the immune system function, is a well-documented phenomenon. The study by Bonanad et al. (2020) [19] analyzed data from over 600,000 patients and showed that older age is associated with higher mortality from COVID-19, which may be the result of a weakened immune response and lack of effective infection control.

At the cellular level, studies such as those by Opitz et al. (2009) [20] and Blasius and Beutler (2010) [21] demonstrate that the endothelium—which also plays an immunological role—undergoes the activation of Toll-like receptors (TLRs), particularly in response to the presence of viral RNA, such as that from SARS-CoV-2. In older individuals, these mechanisms may become dysregulated—both due to the reduced activity of T and B lymphocytes and weakened cytokine production. This phenomenon results in a delayed response to infection.

Interestingly, this weakened immune response might also have a protective effect—older individuals may be less susceptible to excessive inflammatory reactions (cytokine storms), which, in younger patients, often lead to severe COVID-19 complications. This effect requires further investigation. Future studies could include cytokine-level analyses in individuals over the age of 75. Promising results have been observed regarding fluctuations in interleukin-6 levels in patients with acute infectious diseases.

For the purposes of this study, these analyses were not conducted, leaving this an attractive area for future research.

Elderly people often have a decreased immune response, which is associated with immunosenescence—a decrease in T and B lymphocyte activity and decreased interferon production. This may lead not only to a lower reactivity to the SARS-CoV-2 virus but also to a reduction in the cytokine storm, which is often responsible for COVID-19 complications in younger patients. At the same time, the remodeling of blood vessels (e.g., the development of collateral vessels and reduced reactivity of the vessels) may partially compensate for the restriction of blood flow resulting from the infection [22]. Older people with chronic diseases may demonstrate the development of adaptive processes that minimize the risk of sudden cardiovascular complications [23]. A special role is attributed to the remodeling of blood vessels (an observed increase in the level of platelet-derived and fibroblast-derived growth factors) and hemodynamic adaptation in patients with a severe course of COVID-19 [24].

This may be due to compensation mechanisms (increase in blood pressure to maintain perfusion in injured areas, increased vascular stiffness, and decreased reactivity of the renin–angiotensin–aldosterone system). Chronic exposure to cardiovascular stressors may also lead to adaptive mechanisms that are more effective in cases of viral infections than in younger patients with sudden inflammatory reactions [25].

The mutation of the virus may also be expected to impact mortality, as the risk of death was 2.11 times greater in 2021 than in 2022. In 2021, the dominant type was 21A, 21I, 21J (Delta), which was considered more virulent and more often fatal, whereas, in 2022, the type 21K, 21L, 21M (Omicron) was most frequently isolated, which usually caused fewer complications resulting in the death of the patients. It is also possible that the fact that some TH patients were vaccinated against SARS-CoV-2 had a significant impact on the milder course of the disease in 2022. A year earlier, the vaccine had not yet been available.

In the analyzed population, over 23% of the patients did not survive the COVID-19 infection. In patients with a diagnosed cardiovascular pathology, the proportion of deaths was 7% higher compared to the number of study subjects without any cardiac burden. The results obtained are consistent with a study carried out in the US on a large population, the aim of which was to analyze the recovery of the patients after the acute phase of COVID-19 over a 6-month follow-up. Longer recovery times have been observed in participants with pre-pandemic health conditions. After the simultaneous consideration of all pre-pandemic illnesses, only the occurrence of cardiovascular diseases was associated with a difficult recovery [26]. In the same way as in the cited study, no relationship of mortality with the patients’ sex was confirmed.

Similar observations were made by analyzing the course of the SARS-CoV-2 infection in 2020 in a group of over 1000 patients in China. Early reports of COVID-19 cases in China showed that cerebrovascular and cardiovascular diseases were common comorbidities in patients with COVID-19. Further studies have shown that both cerebrovascular and cardiovascular diseases were associated with a higher rate of a severe course of COVID-19, which requires monitoring in the ICU [27].

Similarly, a meta-analysis [28] has shown that patients with cardiovascular disease, hypertension, and acute cardiac injury are more likely to die from COVID-19. Studies by Parohan, Yaghoubi, and Seraji [29] in turn have clarified the link between elevated cardiac injury markers (LDH, CK-MB, CK, and myoglobin) and increased mortality due to COVID-19. Specific biochemical tests were therefore proposed, which enable the prediction of a severe course of SARS-CoV-2 resulting from the coexistence of cardiovascular pathology. Many authors [30], focusing on patients who died of COVID-19, also pointed to a high proportion of patients with co-existing diseases, including hypertension, cardiovascular disease, and diabetes. Increased cardiac injury markers such as troponin I, creatine kinase MB, myoglobin, and NT-proBNP have also been observed in these patients. It is therefore worth drawing attention to the importance of organizing SARS-CoV-2 treatment centers, such as temporary hospitals, in such a way as to enable the performance of extended diagnostic tests, and not to base patient care solely on isolation rooms. The organizational aspect is highlighted, among others, by Borzuchowska et al., analyzing the functioning of health care facilities under pandemic conditions [31].

Research on the physiology of aging in organisms appears to be a highly attractive area of analysis in the context of systemic management of the treatment process, particularly regarding life-threatening diseases.

With age, the circulatory system undergoes numerous adaptations that may have both protective and pathological effects in the context of COVID-19 infection. The study by Zota et al. (2022) [32] indicates that in older individuals, structural changes occur in blood vessels, including the development of collateral circulation, which may compensate for the restricted blood flow caused by infection.

Meanwhile, Rossman et al. (2017) [33] show that endothelial cell aging leads to their senescence, which reduces their ability to produce nitric oxide (NO)—the main mediator of blood vessel dilation. This effect can be partially counteracted by regular physical activity. In the temporary hospital, no data on patient lifestyles were collected. The focus was on the infectious process and its consequences. Pedrinolla et al. (2020) [34] document that the lack of movement (e.g., during hospitalization) further weakens vascular function by worsening endothelial dysfunction. This can result in long-term consequences such as limited vascular reactivity and an increased risk of thromboembolic complications.

Godo and Shimokawa (2017) [35] provide a comprehensive discussion of endothelial function and highlight that its dysfunction not only impairs vascular tone regulation but is also associated with disrupted hemostasis and inflammation, which is particularly dangerous in COVID-19 patients.

As we can see, the image of the aging heart is not unambiguous, and despite the clear risk factors for death, certain defensive mechanisms can be identified within it—mechanisms that could potentially be enhanced pharmacologically.

Among cardiac conditions, authors point to the problem of atrial fibrillation, which is a relatively frequent cause of patients reporting to emergency wards. During the pandemic, the reporting of these patients to hospitals was lower than in the season preceding the mass occurrence of SARS-CoV-2 cases [36]. In turn, the success of cardioversion was statistically more frequent in the pre-pandemic period. One may suspect that the patients, fearing the transmission of coronavirus, gave up obtaining assistance in emergency wards but used it in a more acute phase of the disease. However, in the third wave of the pandemic, patients with a positive test result and an AF/AFL incident were older and more often had newly diagnosed AF/AFL than patients with a negative test result, which indicates the arrhythmogenic impact of the virus in the initial phase of the disease, especially in an older population [37]. The observations we have made are therefore convergent with the conclusions on the interrelation between the severity of the course of COVID-19 pneumonia and cardiovascular system pathologies.

## 6. Limitations

A limitation of this study is the relatively limited population of patients participating in the study. This resulted from the limited duration of the temporary hospital’s operation, as specified by the Minister for Health. In addition, no access was obtained to complete medical data of patients treated in other specialized facilities in Poland. The extension of similar retrospective studies to other institutions could allow for more generalized conclusions to be made.

The analysis of 1244 complete medical records of patients at the temporary hospital in Gdańsk allows us to infer a model of disease progression primarily in the Pomeranian Voivodeship. If similar data compilations were available from across Poland, it would be possible to compare these results with other regions. What would be particularly attractive are comparisons with regions where, due to the location of extractive industries, an increased incidence of respiratory diseases is observed.

At the same time, a multicenter study could confirm or challenge the uniform impact of the analyzed health burdens on mortality. Additionally, enriching the variable pool with anthropometric data could reveal further health correlations (e.g., smoking, obesity, or participation in a vaccination program). However, there would be a risk of incorrect inference regarding the causes and effects of the phenomena described in the material. For example, the risks associated with the development of cardiovascular diseases could be mistakenly classified as directly related to severe COVID-19 progression.

The impact of vaccination on the progression of SARS-CoV-2 infection is a highly complex phenomenon. Observations should include the time interval between vaccination and symptom onset, the number of doses received, and the type of vaccine administered in relation to the immunization schedule. This type of analysis should be the subject of a separate study.

There was also no attempt to refine the analysis by the more precise differentiation of patients depending on specific diagnosis codes (e.g., coronary artery disease, hypertension, atherosclerosis, and ischemic stroke). This was due to the insufficient size of the homogeneous groups of patients, and consequently the difficulty in formulating general conclusions.

## 7. Conclusions

Considering the analysis of available sources and the results of the obtained analyses, a conclusion may be made that cardiovascular disease constitutes a significant factor for the risk of death in patients with COVID-19. Both the presence of co-existing diseases, such as hypertension or coronary artery disease, as well as increased cardiac injury markers (e.g., LDH, CK-MB, and troponin I) correlate with increased mortality in patients with COVID-19.

The patient’s age also modifies the impact of cardiovascular diseases on the prognosis. In this study, the presence of cardiovascular disease in patients under the age of 73 increases the risk of death, while, in older patients, the risk of death paradoxically decreases. The explanation of this unexpected trend requires further research.

COVID-19 patients burdened with cardiovascular diseases require the provision of comprehensive medical care. Early diagnosis, the monitoring of cardiac injury markers, and appropriate treatment may reduce mortality in this group of patients.

Further studies are needed on the impact of COVID-19 on the cardiovascular system. More extensive research is needed, considering the long-term effects of COVID-19 on cardiac health, as well as research on the effectiveness of different therapeutic strategies.

These conclusions highlight the need for special attention and care for COVID-19 patients with cardiovascular diseases. In the face of the COVID-19 pandemic, providing them with the best medical care is crucial for reducing mortality and improving prognosis.

## Figures and Tables

**Table 1:** Detailed classification of cardiovascular disease diagnoses.

ICD-10	Diagnosis Group	Survived	Deaths	Total
Most common:				
I10, I11, I15	Hypertensive disease	521	152	673
I47, I48, I49	Cardiac arrhythmias	133	77	210
I50	Heart failure	106	95	201
I20, I25	Ischemic heart disease	101	45	146
I26	Pulmonary embolism	92	23	115
I63, I64, I65, I67, I69	Stroke and its sequelae, cerebrovascular diseases	67	32	99
Other:				
I70	Atherosclerosis of the aorta and peripheral arteries	52	19	71
I80, I81, I82, I83, I85, I87	Peripheral vein diseases	18	7	25
I07, I08, I09, I34, I35	Valvular defects, endocardial diseases	13	4	17
I21, I22, I23	Acute myocardial infarction	9	1	10
I71	Aortic aneurysm	4	5	9
I42	Cardiomyopathies	6	2	8
I44, I45	Conduction disorders	5	2	7
I46	Cardiac arrest	6	1	7
I73, I74	Peripheral arterial thrombosis and embolism	3	2	5
I30	Acute pericarditis	0	1	1
	Total	1136	468	1604

**Table 2:** Detailed classification of respiratory disease diagnoses.

ICD-10	Diagnosis Group	Survived	Deaths	Total
Most common:				
J12	Viral pneumonia	470	178	648
J96	Respiratory failure	53	108	161
J44	Chronic obstructive pulmonary disease (COPD)	63	25	88
J43, J98	Pulmonary emphysema	25	11	36
J45	Bronchial asthma	32	4	36
Other:				
J16, J18, J22	Other and unspecified pneumonia	18	12	30
J13, J15	Bacterial pneumonia	13	5	18
J93	Pneumothorax	4	5	9
J80	Acute respiratory distress syndrome (ARDS)	0	8	8
J47	Bronchiectasis	6	1	7
J86, J90, J94	Empyema, hemothorax, pleural effusion	3	3	6
J69	Aspiration pneumonia	1	2	3
J81	Pulmonary edema	2	1	3
J20, J21	Acute bronchitis and bronchiolitis	2	0	2
J85	Lung abscess	0	2	2
J02	Acute pharyngitis, unspecified	1	0	1
J05	Acute epiglottitis	1	0	1
J06, J32	Acute laryngitis and pharyngitis	1	0	1
J32	Chronic maxillary sinusitis	1	0	1
J61	Pneumoconiosis	1	0	1
J84	Other specified interstitial lung diseases	1	0	1
	Total	698	365	1063

**Table 3:** Detailed classification of oncological disease diagnoses.

ICD-10	Diagnosis Group	Survived	Deaths	Total
D37–D44	Neoplasm of uncertain or unknown behavior	17	5	22
C91, C94, C95	Hematologic malignancies	9	3	12
C61	Prostate cancer	7	2	9
C34	Bronchial and lung cancer	7	1	8
C50	Breast cancer	6	2	8
C64, C67	Urinary system cancers	5	3	8
C18, C19, C20	Malignant tumor of the colon and rectum	4	3	7
C79	Secondary (metastatic) tumors of bone and nervous system	5	1	6
C15, C16	Malignant tumor of the stomach and esophagus	4	1	5
C78	Other malignant tumors of the digestive tract	2	2	4
C25	Pancreatic cancer	2	1	3
C54, C56	Ovarian and uterine cancers	2	1	3
C82, C85	Non-Hodgkin lymphoma	3	0	3
C31, C32	Cancer of the larynx and ethmoidal cells	1	1	2
C80	Malignant neoplasm, unspecified site	1	1	2
C90	Multiple myeloma	2	0	2
	Total	77	27	104

**Table 4:** Quantitative summary of patients with co-existing pathologies.

Factor	2021 (n = 701)	2022 (n = 543)	*p*-Value
cardiovascular diseases	12.3% (86)	16.4% (89)	0.0381
respiratory pathologies	65.9% (462)	72.1% (392)	0.0178
diabetes	24.8% (174)	24.3% (132)	0.8352
medical history of cancer	5.6% (39)	12.9% (70)	0.0001

**Table 5:** Summary of the number of dead patients meeting the qualitative criteria analyzed.

Analyzed Criterion		Number of Deaths		*p*-Value
		n	%	
year	2021	173/701	24.68%	0.1951
	2022	117/543	21.55%	
sex	women	135/565	23.89%	0.6579
	men	155/679	22.83%	
respiratory pathology	yes	47/175	26.86%	0.2315
	no	243/1069	22.73%	
cardiovascular disease	yes	219/854	25.64%	0.0040
	no	71/390	18.21%	
diabetes	yes	72/306	23.53%	0.9175
	no	218/938	23.24%	
medical history of cancer	yes	32/109	29.36%	0.1181
	no	258/1135	22.73%	

**Table 6:** Univariate logistic regression analysis of odds of death.

No.	Variable	OR	95% CI	*p*-Value
1.	age	1.054	(1.043; 1.066)	<0.001
2.	sex	0.942	(0.724; 1.226)	0.658
3.	year	0.838	(0.642; 1.095)	0.195
4.	length of hospitalization	0.995	(0.980; 1.010)	0.506
5.	cardiovascular diseases	1.550	(1.148; 2.091)	0.004
6.	respiratory system diseases	1.248	(0.868; 1.795)	0.232
7.	diabetes	1.016	(0.749; 1.378)	0.917
8.	cancers in medical history	1.413	(0.914; 2.182)	0.120

**Table 7:** Multivariate logistic regression analysis of odds of death.

No.	Variable	OR	95% CI	*p*-Value
1.	age	1.088	(1.065; 1.112)	<0.001
2.	length of hospitalization	0.979	(0.962; 0.995)	0.011
3.	year	0.474	(0.348; 0.645)	<0.001
4.	cardiovascular diseases	11.557	(1.810; 73.787)	0.010
5.	cardiovascular diseases × age	0.967	(0.943; 0.991)	0.008

**Table 8:** Relationships of predicted to observed values.

Classification		Observed Values	
		Death	Survival
Values predicted	death	237	454
	survival	53	500

## Data Availability

The data are available upon request from the authors.

## Abbreviations

AUC	Area Under The Curve
AF/AFL	Atrial Fibrillation/Atrial Flutter
CI	Confidence Interval
CK-MB	Creatine Kinase–Muscle/Brain isoenzyme
CVD	Cardiovascular Diseases
e.g.,	exempli gratia, for example
LDH	Lactate Dehydrogenase
m	Meter
MB	Myoglobin
NO	Nitric Oxide
NT-proBNP	N-terminal pro B-type Natriuretic Peptide
OR	Odds Ratio
RNA	Ribonucleic Acid
ROC	Receiver Operating Characteristic
SARS	Severe Acute Respiratory Syndrome
Sp. z o. o.	Limited Liability Company
TH	Temporary Hospital
US	United States
